# Light Spatial Distribution in the Canopy and Crop Development in Cotton

**DOI:** 10.1371/journal.pone.0113409

**Published:** 2014-11-19

**Authors:** Xiaoyu Zhi, Yingchun Han, Shuchun Mao, Guoping Wang, Lu Feng, Beifang Yang, Zhengyi Fan, Wenli Du, Jianhua Lu, Yabing Li

**Affiliations:** Institute of Cotton Research of the Chinese Academy of Agricultural Sciences/State Key Laboratory of Cotton Biology, Anyang, 455000, Henan, China; University of Vigo, Spain

## Abstract

The partitioning of light is very difficult to assess, especially in discontinuous or irregular canopies. The aim of the present study was to analyze the spatial distribution of photosynthetically active radiation (PAR) in a heterogeneous cotton canopy based on a geo-statistical sampling method. Field experiments were conducted in 2011 and 2012 in Anyang, Henan, China. Field plots were arranged in a randomized block design with the main plot factor representing the plant density. There were 3 replications and 6 densities used in every replicate. The six plant density treatments were 15,000, 33,000, 51,000, 69,000, 87,000 and 105,000 plants ha^−1^. The following results were observed: 1) transmission within the canopy decreased with increasing density and significantly decreased from the top to the bottom of the canopy, but the greatest decreases were observed in the middle layers of the canopy on the vertical axis and closing to the rows along the horizontal axis; 2) the transmitted PAR (TPAR) of 6 different cotton populations decreased slowly and then increased slightly as the leaves matured, the TPAR values were approximately 52.6–84.9% (2011) and 42.7–78.8% (2012) during the early cotton developmental stage, and were 33.9–60.0% (2011) and 34.5–61.8% (2012) during the flowering stage; 3) the Leaf area index (LAI) was highly significant exponentially correlated (R^2^ = 0.90 in 2011, R^2^ = 0.91 in 2012) with the intercepted PAR (IPAR) within the canopy; 4) and a highly significant linear correlation (R^2^ = 0.92 in 2011, R^2^ = 0.96 in 2012) was observed between the accumulated IPAR and the biomass. Our findings will aid researchers to improve radiation-use efficiency by optimizing the ideotype for cotton canopy architecture based on light spatial distribution characteristics.

## Introduction

Crop yields depend on a canopy's capacity to intercept and efficiently use solar radiation. Photosynthetically active radiation (PAR) represents the solar radiation that can be absorbed by green plants [1] and used for photosynthesis to produce biomass [2]–[5]. Canopy architectural information is essential to a mechanistic description of radiation interception [6].

In 1953, Beer's law [7] was used to measure the leaf area and light intensity within each layer based on height in order to describe the spatial distribution of light. Since then, numerous investigations of radiation interception have been conducted using various approaches [8]–[11] and models such as CERES [12]–[13], GROPGRO [14], AFRCWHEAT [15] and CropSyst [16] based on their description of light extinction in plant canopies. Rosenthal [17] argued that the crop extinction coefficient had a negative linear relationship with the leaf area index (LAI). Campbell [18] expressed the extinction coefficient of the population using a function based on the angle of the sun and the leaf angle distribution. However, the crop population extinction coefficient is a variable that is sensitive to environmental factors and is difficult to measure. Furthermore, previous researchers [19]–[20] argued that Beer's law failed to fully consider the canopy spatial heterogeneity, which has often led to discrepancies between models and experimental data. Therefore, the application of these methods to estimate light distribution remains limited, and a more complete model is required.

Muchow et al. [21] suggested using four tube solarimeters in each plot to obtain estimates of radiation interception for sugarcane (*Saccharum officinarum*). Alados [22] studied measurements of solar global irradiance using a Kipp & Zonen model CM-11 solarimeter (Delft, Netherlands), and Singer et al. [23] measured the cumulatively intercepted PAR by deploying eight line quantum sensors in each of their experimental fields; however, the partitioning of light in different density systems is very difficult to assess, especially in discontinuous or irregular canopies. In theory, an accurate assessment could be achieved by placing a large number of sensors across the canopy to cope with spatial variability, but this solution is impractical due to the increased labor and capital costs involved [24]–[25]. Furthermore, Campillo et al. [26] used digital images and line quantum sensors to characterize light interception, which neglected the canopy architecture's effect on the spatial distribution of light. Previous research has focused on improving the efficiency of light utilization and exploring the spatial distribution of light [25], [27]–[29]. Light interception has commonly been measured with expensive equipment or estimated with elaborate models; therefore, simpler and more economical methods, particularly techniques that consider spatial heterogeneity, are highly desirable. In addition, the different light intensities caused by different cotton plant densities have not yet been determined [30]–[31]. However, some studies have indicated that leaf area components had the greatest effect on light intensities [32], and Yang [33] concluded that different cotton canopy structures caused different light intensities. To determine the optimal plant density for biomass production, it is crucial to determine the spatial distribution of light in more detail.

Spatial heterogeneity effects should be considered in the study of light distribution characteristics in a canopy. The distribution of PAR in plant canopies is influenced not only by the radiation intensity but also by the plant density [34]–[38], the leaf angle [39]–[41], the nutritional status [3], [42], and the LAI [3], [35], [43]–[46]. Spatial statistics are a versatile tool for environmental disciplines such as agriculture, geology, soil science, hydrology, ecology, oceanography, forestry, meteorology and climatology [35], [47]–[49].

The objectives of this study were as follows: 1) to quantify the spatial distribution of light in heterogeneous cotton canopies; 2) and to explore the PAR variation and distribution characteristics under different plant densities using a geo-statistical sampling method to provide the theoretical and technical basis for optimizing the canopy architecture to intercept more radiation and improve the cotton lint yield.

## Materials and Methods

### 1 Experimental design

Field assays were conducted in 2011 and 2012 at the Cotton Research Institute of the Chinese Academy of Agricultural Sciences in Anyang, Henan, China (36° 06 ′N, 114° 21′ E). During the cotton developmental stage, the average temperature was 21.2°C in 2011 and 23.2°C in 2012, and the total rainfall was 448.1 mm in 2011 and 421.3 mm in 2012. The same field was used in each year and was characterized by a medium loam soil with a total N 0.66 g kg^−1^, P 0.01 g kg^−1^ and K 0.11 g kg^−1^. The land was plowed and irrigated in early spring before planting. A randomized block design was used with 6 treatments and 3 replicates. The area of every plot was 64.0 m^2^ with 8.0 m width, 8.0 m length and 0.8 m row spacing. The 6 plant density treatments were 15,000, 33,000, 51,000, 69,000, 87,000 and 105,000 plants ha^−1^. The plants were sown by machine on April 19, 2011 and April 18, 2012. The sampling areas were free of weeds, and all of the plots received fertilizer at 225.0 kg ha^−1^ N, 150.0 kg ha^−1^ P_2_O_5_ and 225.0 kg ha^−1^ K_2_O. Irrigation was applied at a volume of approximately 40 m^3^ in total by flooding the furrows during the flowering stage. Weeds were manually controlled; pesticides were used to control insects and diseases.

### 2 Collection of PAR data

The incident PAR (PARi), PAR reflection (PARr) and agronomic characters (green leaf area, dry mass, boll weight and lint yield) were measured every ten days. The fluxes of PARi and PARr at each layer were measured with a spatial grid method [50], and the incident PAR at 20 cm (PARI) above the canopy was measured synchronously. The PARi and PARr between 2 rows of each plot were measured under clear or partly cloudy conditions at 10:00 am using a 100 cm line light quantum sensor (LI-191SA, LI-COR, Lincoln, NE, USA) and datalogger (LI-1400, LI-COR, Lincoln, NE, USA). The canopy was divided into 6 or 7 thin vertical layers according to plant height and 5 horizontal layers [51]. Then the PARi and PARr of 30 or 35 positions within the cotton canopy were measured. The volume within an area of 100 cm of row length and between 2 rows 80 cm apart up to a height of 100 cm was sampled every 10 days. The sensor was placed parallel to the row orientation and measured the light above a row at 100 cm, then moved 20 cm towards the adjacent row and measured the light again; this was performed again at 40 and 60 cm before being placed above the adjacent row (80 cm from the initial row). The instrument was then lowered to 80 cm and the process was repeated at 60, 40, 20 and 0 cm above the ground to provide a comprehensive set of spatial data of light intensities within the canopy. This process was repeated every 10 days, but only the data from 2 dates in each year is reported here. The model calculated, by interpolation, contours lines of equal light intensity. The canopy TPAR, RPAR and IPAR were calculated using the following equations [52]: 
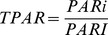
(1)

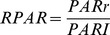
(2)

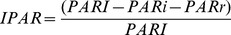
(3)where PARI is the incident PAR at 20 cm above the canopy (µmol·m^−2^·s^−1^), and PARi and PARr are the incident PAR and PAR reflection at each layer of the canopy, respectively.

### 3 Estimating cotton canopy PAR

The PARi, PARI and PARr in other positions in the canopy were calculated. Value estimates were calculated by spatial interpolation as follows:
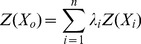
(4)where *Z(x_0_)*  = measured PAR values, *λ_i_* = the coefficient of the sample, and the unbiased condition *∑λ_i_* = 1 was employed. Based on the minimum variance, the Kriging equation [53]–[54] is stated as follows:



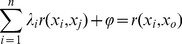
(5)Where *φ* =  Lagrangian, *r (x_i_, x_j_)*  =  the measured value of the variation function, *r (x_i_, x_0_)*  =  the measured and calculated PAR, and *x_0_* is the estimated value of the calculated point as computed by the unbiased estimate.

The TPAR within the canopy was computed by the Simpson 3/8 rules [55]. Surfer software V12 (Golden Software Inc., USA) was used with the application of the following equation (6):
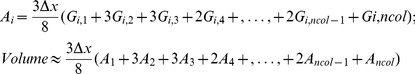
(6)where the coefficient vector is *[5,3,3,2,…, 3,3,2,1]*, Δ*x* is the vertical distance of the grid, Δ*y* is the horizontal distance; *G (I, j)* is the grid node number (*I, j*), and volume is the total light volume of a certain cross-sectional area.

### 4 Agronomic traits of cotton

Two randomly selected plants from each plot were harvested every 10 days in 2011 and 2012. During sampling, at least two edge rows were excluded to avoid the boundary effects. These destructive samples were subdivided into leaves, stems, flowers and bolls depending on their developmental stage. The leaf area was determined using a scanner (Phantom 9800xl, MiCROTEK, Shanghai, China) [56] and was measured using Image-Pro Plus (Media Cybernetics, Inc.). After the leaf areas were measured, the leaves, stems and bolls were dried at 80°C to a constant weight, and the dry mass was determined.

## Results

### 1 Cotton PAR of the entire canopy throughout the cotton growth period

**Figure 1:**
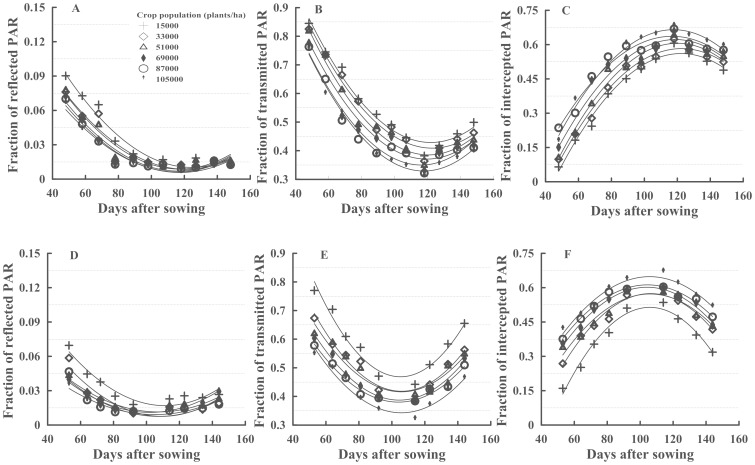
The variation of reflected, transmitted and intercepted PAR of all the plant densities over the growing period of cotton in 2011 (A, B, C) and 2012 (D, E, F).

To study the PAR spatial distribution, the values of the TPAR, RPAR and IPAR were calculated using the 3/8 Simpson and Quadratic relationships of the days after sowing for the PAR (Table 1, Fig. 1) with highly significant and determination coefficients all above 0.90. Furthermore, the values of “A” were positive for the TPAR and RPAR simulation equations and negative for the IPAR simulation equations (Table 1).

**Table 1:** Simulation equations of transmitted PAR, reflected PAR and intercepted PAR: y = Ax^2^+Bx+C.

Treatments (plants ha^−1^)		2011, n = 11					2012, n = 9				
		A	B	C	R^2^	P>F	A	B	C	R^2^	P>F
PAR transmittance	15000	1.608	−0.019	0.793*10^−4^	0.979	<0.001	1.819	−0.026	0.122*10^−3^	0.950	<0.001
	33000	1.577	−0.019	0.775*10^−4^	0.978	<0.001	1.508	−0.021	0.973*10^−4^	0.929	<0.001
	51000	1.593	−0.020	0.831*10^−4^	0.972	<0.001	1.373	−0.018	0.855*10^−4^	0.893	0.001
	69000	1.515	−0.019	0.803*10^−4^	0.934	<0.001	1.333	−0.018	0.867*10^−4^	0.975	<0.001
	87000	1.487	−0.020	0.844*10^−4^	0.951	<0.001	1.223	−0.016	0.779*10^−3^	0.972	<0.001
	105000	1.192	−0.020	0.840*10^−4^	0.984	<0.001	1.255	−0.017	0.816*10^−4^	0.936	<0.001
PAR reflectivity	15000	0.226	−0.004	0.140*10^−4^	0.961	<0.001	0.189	−0.003	0.137*10^−4^	0.910	<0.001
	33000	0.190	−0.003	0.121*10^−4^	0.907	<0.001	0.166	−0.003	0.132*10^−4^	0.845	0.004
	51000	0.196	−0.003	0.138*10^−4^	0.947	<0.001	0.123	−0.002	0.097*10^−4^	0.917	<0.001
	69000	0.183	−0.003	0.130*10^−4^	0.936	<0.001	0.124	−0.002	0.108*10^−4^	0.925	<0.001
	87000	0.176	−0.003	0.126*10^−4^	0.930	<0.001	0.130	−0.002	0.106*10^−4^	0.808	0.007
	105000	0.163	−0.003	0.114*10^−4^	0.944	<0.001	0.102	−0.002	0.084*10^−4^	0.920	<0.001
PAR interception	15000	−0.835	0.023	−13.330	0.984	<0.001	−1.008	0.029	−4.360	0.969	<0.001
	33000	−0.766	0.022	−12.980	0.982	<0.001	−0.674	0.023	−4.110	0.959	<0.001
	51000	−0.789	0.023	−13.670	0.975	<0.001	−0.496	0.020	−13.520	0.926	<0.001
	69000	−0.697	0.022	−9.3E−05	0.941	<0.001	−0.458	0.020	−13.750	0.980	<0.001
	87000	−0.521	0.020	−12.490	0.951	<0.001	−0.353	0.018	−12.860	0.976	<0.001
	105000	−0.656	0.022	−13.550	0.986	<0.001	−0.357	0.019	−13.000	0.955	<0.001

The TPAR of 6 treatments in 2 years presented quadratic tendencies, with highly significant correlation coefficients between 0.93–0.98 (Table 1). Over the cotton developmental stage, the TPAR values were approximately 52.6–84.9% (2011) and 47.7–78.8% (2012) before 64 days after sowing and were 33.9–60.0% (2011) and 34.5–61.8% (2012) during 65–120 days after sowing (Fig. 1). At the same development stage, the TPAR in 2012 was higher than in 2011, which demonstrated that cotton developed better in 2011 than in 2012. According to Table 1, the minimum TPARs of the different plant densities were 43.0, 41.0, 39.0, 37.0, 35.0 and 33.0% at 122, 123, 121, 119, 116 and 118 days after sowing in 2011, respectively. The minimum TPARs were 47.0, 42.0, 42.0, 39.0, 38.0 and 34.0% at 105, 106, 106, 104, 104 and 106 days after sowing in 2012, respectively.

The RPAR spatial distribution is shown in Fig. 1. The RPAR group declined rapidly and then increased slowly with time. The RPAR was approximately 9.0% in the early days after sowing and 1.0–2.0% in the late days after sowing in 2011. The RPAR of different plant densities decreased with the increase of plant density, particularly in the early developmental stage. At 48 days after sowing in 2011, the RPARs of the 6 different plant densities were 9.0, 7.6, 7.8, 7.3, 7.0 and 6.6% at 15,000, 33,000, 51,000, 69,000, 87,000 and 105,000 plants ha^−1^, respectively. The minimum estimations of RPAR (Table 1) were 1.0% at 124 days after sowing at 15,000 plants ha^−1^ and 0.8% at 121 days after sowing at 33,000 plants ha^−1^ in 2011. In 2011, the RPARs of the other 4 plant densities were 0.9, 0.7, 0.6 and 0.6% at 117, 116, 116, and 117 days after sowing, respectively. In both years, the RPARs of different plant densities were 2.0–6.5% at 64 days after sowing. However, cotton senescence occurred later in 2011 than in 2012; therefore, the RPAR was higher in 2012 than in 2011 during the late developmental stage. In 2012, the minimum RPARs of different plant densities were 1.7, 0.7, 1.1, 1.1, 0.8 and 0.9%, at 111, 109, 107, 102, 107 and 105 days after sowing, respectively.

The IPAR spatial distribution was shown in Fig. 1. The IPAR of different plant densities was 10.0–70.0% over the cotton developmental stage. The maximum estimations of IPAR (Table 1) were 56.0, 58.0, 61.0, 62.0, 64.0 and 67.0% at 122, 123, 120, 119, 117 and 118 days after sowing in 2011, respectively. The maximum IPARs were 51.0, 57.0, 57.0, 60.0, 61.0 and 65.0% at 106, 106,106,104,104 and 106 days after sowing in 2012, respectively. The maximum IPAR in the different plant densities increased with the increase of plant density in both years (Fig. 1), but the difference in quadratic tendencies between the 2 years was caused by excessive cotton vegetative growth in 2012.

### 2 Spatial distribution of the transmitted PAR within the cotton canopy

**Figure 2:**
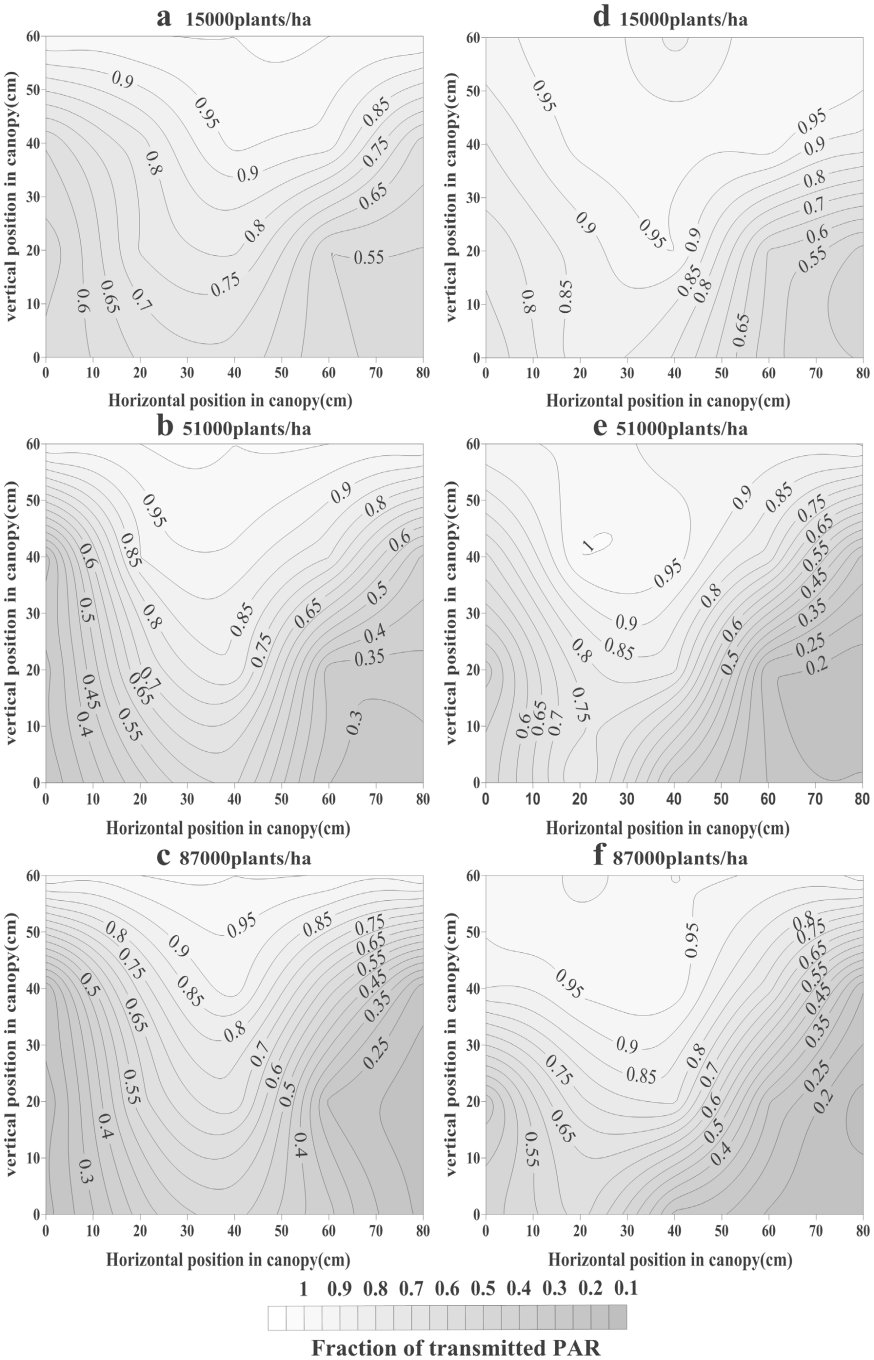
Vertical and horizontal distribution of TPAR at the early cotton developmental stage in 2011 (a, b, c) and 2012 (d, e, f).

**Figure 3:**
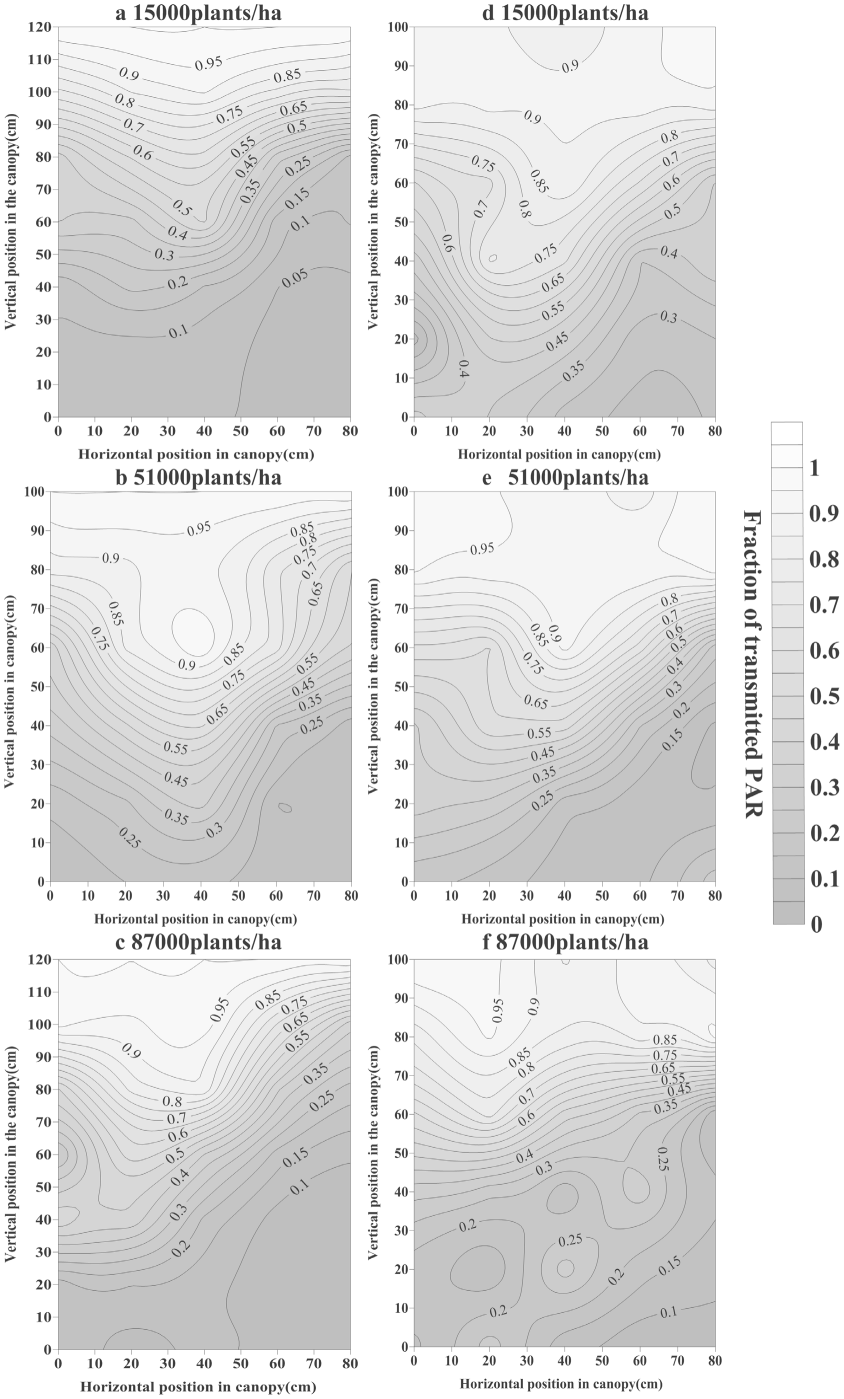
Vertical and horizontal distribution of TPAR at the flowering stage in 2011 (a, b, c) and 2012 (d, e, f).

This study analyzed the TPAR spatial distribution in two developmental stages (Figs. 2 and 3). A tendency toward less TPAR was observed in higher plant densities. For example, at the early cotton developmental stage in 2011 (Fig. 2), from 0–50 cm vertical position, the TPAR was approximately 60.0–80.0% (15,000 plants ha^−1^), 35.0–65.0% (51,000 plants ha^−1^) and 30.0–60.0% (87,000 plants ha^−1^) near the cotton rows (10 cm horizontal position); and was 67.0–95.0% (15,000 plants ha^−1^), 61.0–95.0% (51,000 plants ha^−1^) and 53.0–93.0% (87,000 plants ha^−1^) at the mid-point between rows (40 cm horizontal position). At the flowering stage in 2011 (Fig. 3), from 0–80 cm vertical position, the TPAR was 7.0–45.0% (15,000 plants ha^−1^), 17.0–85.0% (51,000 plants ha^−1^) and 10.0–65.0% (87,000 plants ha^−1^) near the rows (10 cm horizontal position); and 6.0–62.0% (15,000 plants ha^−1^), 22.0–100.0% (51,000 plants ha^−1^) and 12.0–80.0% (87,000 plants ha^−1^) at the mid-point between 2 rows (40 cm horizontal position).

Cotton branch development and leaf area expansion played prominent roles in explaining the TPAR spatial distribution during different years and in different spatial positions. For example, in the early cotton developmental stage (Fig. 2), at a density of 51,000 plants ha^-1^, from 0–80 cm horizontal position, the TPAR ranged from 25.9 to 60.9% at the 0 cm vertical position, but the TPAR was a constant 100.0% at the 60 cm vertical position; however, from 0–60 cm vertical position, the TPAR decreased from 100.0 to 25.6% at the 0 cm horizontal position and to 60.9% at the 40 cm horizontal position in 2011. And at 51,000 plants ha^−1^ density, the TPAR ranged from 20.8 to 76.0% at the 0 cm vertical position and from 91.3 to 95.8% at the 60 cm vertical position; however, the TPAR decreased from 93.8 to 42.4% at the 0 cm horizontal position and from 97.7 to 41.9% at the 40 cm horizontal position in 2012.

### 3 Relationship between the intercepted PAR and leaf area index

**Figure 4:**
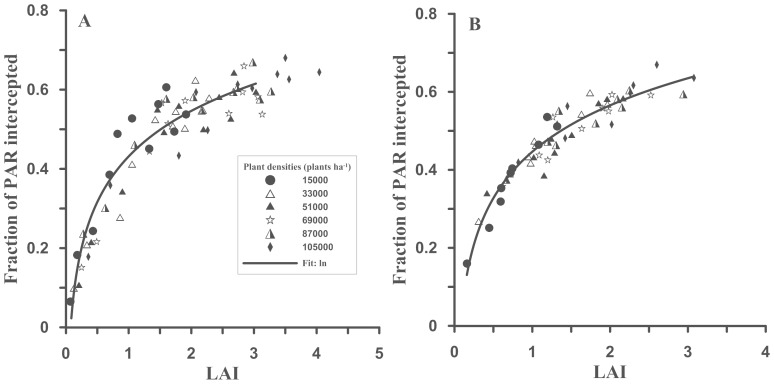
Relationship between the LAI and IPAR in 2011 (A) and 2012 (B).

The LAI exhibited a highly significant logarithmic correlation with the IPAR of different plant densities in both years (2011, n = 66, R^2^ = 0.90, P>|t|: <0.001; 2012, n = 54, R^2^ = 0.91, P>|t|: <0.001) (Fig. 4).
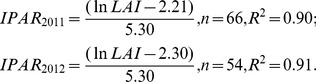
(7)


### 4 Relationship between the intercepted PAR and biomass

**Figure 5:**
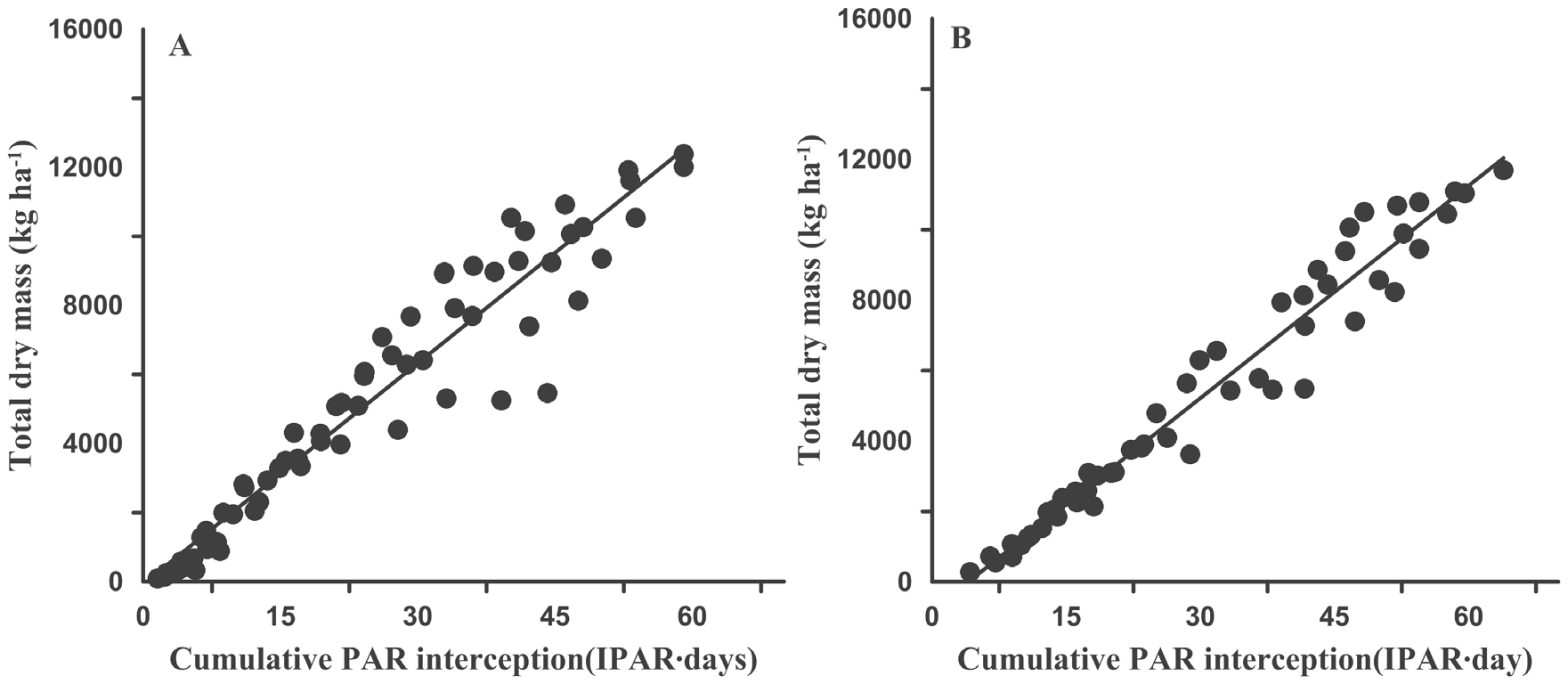
Relationship and fitted models between the cumulative IPAR and dry mass in 2011 (A) and 2012 (B).

Across all plant densities, dry mass accumulation was linearly related to the cumulative IPAR in both years, employing all of the data from different populations and stages into two study years (2011, n = 66, P>F: <0.001; 2012, n = 54, P>F: <0.001) (Fig. 5). 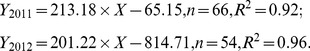
(8)


## Discussion

The results of this work demonstrate that both the TPAR and the vertical distribution of the PAR in the canopy are very important for crop photosynthesis, which is in agreement with previous research [57]. The present study used the grid method to detect existing spatial heterogeneities, and geo-statistics were applied to create contour maps for variables within the canopies. Baldocchi et al. [39] reported a spherical distribution in a canopy radiation transfer model. Subsequent studies by Campbell [58] and Campbell and Norman [59] conducted accurate experiments on a theoretical ellipsoidal distribution model, but validations of this model have been predominantly conducted in herbaceous crops. Canopy architecture influenced the spatial heterogeneity of the PAR in the 6 densities over the study periods, in agreement with the findings of Reta-Sanchez et al. [60] and Stewart et al. [61], who showed that the canopy architecture changes affected light penetration into the canopy. The PAR and canopy architecture determined for cotton in this experiment are in good agreement with the results of Sassenrath-Cole [62], who reported that the extreme cupping of cotton leaves may further increase the photosynthetically active area. Detailed canopy light distribution had been previously shown to improve the total canopy photosynthesis and yield [63].

### 1 Cotton PAR of the entire canopy

The TPAR and RPAR decreased rapidly for light sheltered by the cotton plant in the early developmental stage; however, it increased slowly in the later stage because of leaf senescence. Similarly, Cooper [64] reported a significant correlation between the crop growth rate and the canopy extinction coefficient. In the flowering stage, the horizontal differences between the TPAR were small in the 0 cm vertical position but markedly increased at 40 cm. The TPAR ranged from 11.2% to 38.3% at the 0 cm vertical position but from 13.0% to 62.6% at the 40 cm vertical position. This result might be related to the fact that the lower canopy layers predominantly receive diffuse radiation, which is in general more homogeneously distributed within the canopy.

### 2 Relationship between the intercepted PAR and leaf area index (LAI)

Morris [65] found that the vertically oriented leaves at the top of the canopy intercepted less light than did the horizontal leaves, which is consistent with present research on canopy architecture. In this study, the IPAR was highest at the tops of the canopies, which was in strong agreement with the results of both Sakamoto and Shaw [66] and Hatfield and Carlson [67], who found that approximately 90.0% of light interception occurred in the top and peripheral sections of the canopy. One possible explanation for this result is that the LAI was reduced due to the abscission of lower canopy leaves exposed to shade. When the LAI was at its maximum for all of the plant densities, the differences in the IPAR were unrelated to differences in the maximum LAI [35], which might be due to self-shading of the lower canopy leaves. Tharakan et al. [68] indicated that the LAI and canopy duration were very important for biomass production, which was supported by the findings of the present study.

### 3 Relationship between the intercepted PAR and biomass

The present research identified a close relationship between the biomass and IPAR within the canopy, and biomass was an appropriate index for assessing the IPAR, which was in agreement with the results from Russell et al. [69] and Kiniry et al. [70]. Additionally, Robinson et al. [71] showed that the leaf extension rate and cell number per leaf determined the final leaf size, which in turn influenced the IPAR and yield. However, the IPAR did not correspondingly increase with increased biomass, especially after canopy closure [37], [72]; and this finding was consistent with the results reported by Ceotto et al. [73]. A linear relationship was found between the cumulative IPAR and the biomass, which was in agreement with the results from Christensen [74].

## Conclusions

The results indicated the following: 1) the cotton canopy architecture affected the spatial distribution of the TPAR; 2) the distribution discrepancy was larger at the top of the canopy than at the bottom in the flowering stage; 3) the TPAR variation of the minimum and maximum densities was smaller than those of the other four densities; 4) the IPAR was affected by the LAI for different canopy structure; 5) and the biomass was highly correlated with the IPAR.

The amount and distribution of the leaf area in a crop canopy determined the way that the PAR was intercepted and consequently influenced the canopy photosynthesis and yield. This study analyzed the PAR spatial distribution within the cotton canopy and the relationship between the LAI and IPAR to assess the importance of the IPAR in explaining biomass variation and identifying the optimal canopy structure from a suite of leaf measurements and plant density.

The results of this study will assist breeding researchers in the selection of cultivars with more erect leaves, especially at the top of the canopy, to improve light environments within canopies and canopy photosynthesis [75]. A desirable ideotype will include a more open structure with greater IPAR, leading to increased photosynthesis and yield. Modern developments in cotton breeding can use PAR spatial distribution information to produce more efficient genotypes for canopy photosynthesis thus increasing lint yield. The results of this study may be used to further map the PAR in spatially heterogeneous canopies using geo-statistical interpolation methods, thereby contributing to the development of an ideal plant shape and the breeding of high light use efficiency crops.
